# Different mechanisms of two anti-anthrax protective antigen antibodies and function comparison between them

**DOI:** 10.1186/s12879-019-4508-z

**Published:** 2019-11-07

**Authors:** Siping Xiong, Tingting Zhou, Feng Zheng, Xudong Liang, Yongping Cao, Chunhui Wang, Zhengqin Feng, Qi Tang, Jin Zhu

**Affiliations:** 1 Epidemiological Department, Huadong Medical Institute of Biotechniques, Nanjing, 210002 China; 2 Key Laboratory of Antibody Technique of Ministry of Health, Nanjing Medical University, Nanjing, 210029 China; 3 Department of Pathology, The Eighth Affiliated Hospital of Sun Yat-sen University, Shenzhen, 518033 Guangdong China; 4 National Institute for Communicable Disease Control and Prevention, Chinese Center for Disease Control and Prevention, Beijing, 102206 China

**Keywords:** Anthrax, Protective antigen, Lethal toxin, Neutralizing antibody, Mechanism

## Abstract

**Background:**

*Bacillus anthracis* causes a highly lethal infectious disease primarily due to toxin-mediated injury. Antibiotics are no longer effective to treat the accumulation of anthrax toxin, thereby new strategies of antibody treatment are essential. Two anti- anthrax protective antigen (PA) antibodies, hmPA6 and PA21, have been reported by our lab previously.

**Methods:**

The mechanisms of the two antibodies were elucidated by Electrophoresis, Competitive Enzyme-linked immune sorbent assay, Western blot analysis and immunoprecipitation test, and in vitro*,* in vivo (F344 rats) treatment test. The epitopes of the two antibodies were proved by Western blot and Enzyme-linked immune sorbent assay with different domains of PA.

**Results:**

In this study, we compared affinity and neutralization of these two antibodies. PA21 was better in protecting cells and rats, whereas hmPA6 had higher affinity. Furthermore, the neutralization mechanisms of the two antibodies and their recognition domains of PA were studied. The results showed that hmPA6 recognized domain IV, thus PA could not bind to cell receptors. Conversely, PA21 recognized domain II, thereby limiting heptamer oligomerization of PA63 in cells.

**Conclusions:**

Our studies elucidated the mechanisms and epitopes of hmPA6 and PA21. The present investigation can advance future use of the two antibodies in anthrax treatment or prophylaxis, and potentially as a combination treatment as the antibodies target different epitopes.

## Background

*Bacillus anthracis* is a sporulating Gram-positive bacterium that can cause high morbidity and mortality, and it is also considered as a potential weapon of bioterrorism [1, 2]. In some parts of the world, this lethal disease is still endemic principally to herbivores and also can affect other species, including humans [3]. In the past decade, some terrorists used the anthrax agents and/or their associated toxins as bioweapons. In addition, some people have been exposed to anthrax spores during bioterrorism events [3, 4]. These make it necessary to study anthrax pathogenesis, treatment, etc.

The pathogenesis of *B. anthracis* is mainly caused by anthrax toxin which is a tripartite protein complex. The three-protein toxin consist of a cellular receptors binding component, the protective antigen (PA), and two catalytic components, lethal factor (LF) and edema factor (EF) [5, 6]. First, PA binds to cell surface receptors (the tumor endothelial marker 8, TEM-8; the capillary morphogenesis protein-2, CMG-2) [7, 8]. Subsequently, the amino-terminal 20-kDa region of PA is cleaved by furin protease and released. The remaining portion of PA bound on cell surface, named PA63, forms a homo-heptamer, which can bind and transduce EF/LF into cells. LF is a zinc-dependent protease specific for the mitogen-activated protein kinase family, and EF is a calmodulin-activated adenylyl cyclase [9–11]. Therefore, LF or EF could induce cells lethal or edema effect separately.

Although, at the early stages of anthrax, antibiotics can be effective for bacterial elimination [12, 13]. With the accumulation of anthrax toxin, antibiotics are no longer effective and the disease is often lethal despite treatment [14]. Thus, at later stages of anthrax, other countermeasures are essential. Therefore, several studies, mainly focus on PA, LF, or capsular antigen, have been searching for various therapeutic strategies [15–17]. As such, the most promising approach employed anti-toxin antibody treatment to generate a state of immediate passive immunity.

In our previous studies, we developed two anti-PA antibodies that showed good function in neutralizing lethal toxin [18, 19]. Therefore, we studied the neutralization mechanisms of these two antibodies. According to the pathogenesis, PA is divided into four domains: domain I (residues 1–258) contains the furin proteolysis site, and the furin proteolysis site make domain I to domain I a and domain I b (domain I b explores the LF/EF binding site); domain II (residues 259–487) and domain III (residues 488–595) are involved in heptamer and pore formation; domain IV (residues 596–735) binds to the cellular anthrax toxin receptors [20]. Here, we compared these two antibodies on aspects of affinity and protective function. Further, we reported their neutralization mechanisms based on the pathogenesis and characterize which domain they recognize.

## Methods

### Affinity and neutralization assay

The affinity, in vitro and in vivo neutralization assay of hmPA6 and PA21 were reported by our lab previously [18, 19]. Briefly, the affinity was detected by Biacore X100. The in vitro neutralization assay was performed with J774A.1 cells which were incubated with lethal toxin and antibodies. The in vivo neutralization assay was performed with F344 rats which were injected with lethal toxin and antibodies in different time points via tail vein.

### Interference with LF binding

#### Competitive ELISA

The enzyme immunoassay plates were coated with 100 μL PA63 antigen at a concentration of 2 μg/mL overnight at 4 °C. The PA63 was diluted in 50 mM sodium carbonate buffer (pH 9.6). After blocking, serial two-fold dilutions of LF and 0.125 μg/mL PA21 or 0.0625 μg/mL hmPA6 were added to the wells (3 wells for each concentration) with 2 h incubation at 37 °C. Then the experiment was done as previous described ELISA [19]. The experiment was done for three independent times.

#### Co-immunoprecipitation (co-IP)

A mixture of PA63 and LF were incubated with different amounts of hmPA6 at 4 °C and rotated for 3 h. Next, 50 μL protein-A Sepharose (Invitrogen, USA) was added and incubated at 4 °C. The immune complexes that formed were washed three times with PBST. Subsequently, 50 μL elution buffer was added to separate these antibody-antigen complexes from protein-A Sepharose. As a negative control, LF was incubated with hmPA6 alone. The protein complexes were isolated by running two 10% SDS-PAGE gels; and they were transferred onto a nitrocellulose membrane. The nitrocellulose membranes were blocked at 4 °C overnight, one incubated with 1:5000 diluted rabbit polyclonal anti-PA antibody (Pierce, USA) and the other incubated with anti-LF antibody for 1 h at RT, washed with PBST 3 times, and reacted with 1:4000 diluted goat anti-rabbit IgG-HRP conjugate (Sigma,USA) for an additional 30 min at room temperature. The membrane was washed 3 times with PBST, and the hybridization signal was detected using ECL Western Blot substrate (Millipore, USA). Similar procedures were used for PA21 detection.

#### Neutralization assay of cell and animal

The assay was performed as previously described [19], with slight modification that the antibody, PA and LF were treated separately. In cell neutralizing assay, PA was added 3 h before the mixture of LF and different amount of antibody. The experiment was performed independently 3 times.

F344 rats weighing between 130and 160 g were ordered from Charles river Company (Beijing, China). 24 female rats were divided into 4 groups, with 6 rats in each group. PA was administered before or after the mixture of LF and antibody. The alive rats in each group will be record. The remaining alive rats will be euthanized by carbon dioxide. All experiments involving animals were performed in accordance with the protocols approved by the Animal Care and Use Committee of the Nanjing Medical University, China.

### Inhibition of PA binding to receptor——Western blot

The murine macrophage J774A.1 cells owned by our lab were cultured in 24-well plates overnight. One microgram PA83 and increasing amounts of antibody were added to wells. After 3 h incubation, the wells were washed with PBS three times. For cell lysate, RIPA (Radio Immunoprecipitation Assay) lysis buffer (Promab) was added to cells and incubated for 30 min on ice. The cell lysates were isolated by 10% SDS-PAGE. The following western blot experiment was done as previously described [18]. The PA binding to cells was detected, and GAPDH served as a control.

### Interference with Furin cleavage

Briefly, 25 μg PA was incubated in 100 μL PBS with 1-unit Furin (Sigma) at RT for different durations. According to this, 25 μg PA and different amounts of antibody were incubated in 100 μL PBS with 1-unit Furin at RT for 6 h. Following, the mixture was isolated by running on 10% SDS-PAGE gels and stained with Coomassie Blue.

### Mapping of MAb binding to PA

The individual protein domains of PA, which are composed of amino acids 1 to 258 (domain I), 596 to 735 (domain IV), 164 to 487 (domainIb, II), and 164 to 595 (domain Ib, II and III), were cloned into *Escherichia coli* strain BL21, expressed, and purified as described previously [21]. Each recombinant domain was resolved by SDSPAGE and transferred to nitrocellulose for immunoblotting. The blots were blocked with 5% nonfat dry milk in 10 mM Tris-buffered saline (PBS), pH 7.3 and then probed with each monoclonal antibody at an appropriate dilution. The remaining steps was performed as Western blot section described. The domain 4 was also performed by ELISA. The procedure was conducted as described before.

### Statistical analysis of survival data

Kaplan Meier analysis was used for evaluation of survival. Survival data were analyzed using the GraphPad Prism version 5 statistical analysis software (San Diego, CA). A two-tailed log rank test was used to determine the statistical significance of differences between groups. A *P* value of < 0.05 was considered statistically significant.

Other values are presented as Means ± SEM. One-way or two-way analysis of variance followed by Bonferroni Significant Difference test was used to analyze the differences within groups where appropriate. Significance level was set at *p* < 0.05.

## Results

### Affinity and neutralization

**Fig. 1:**
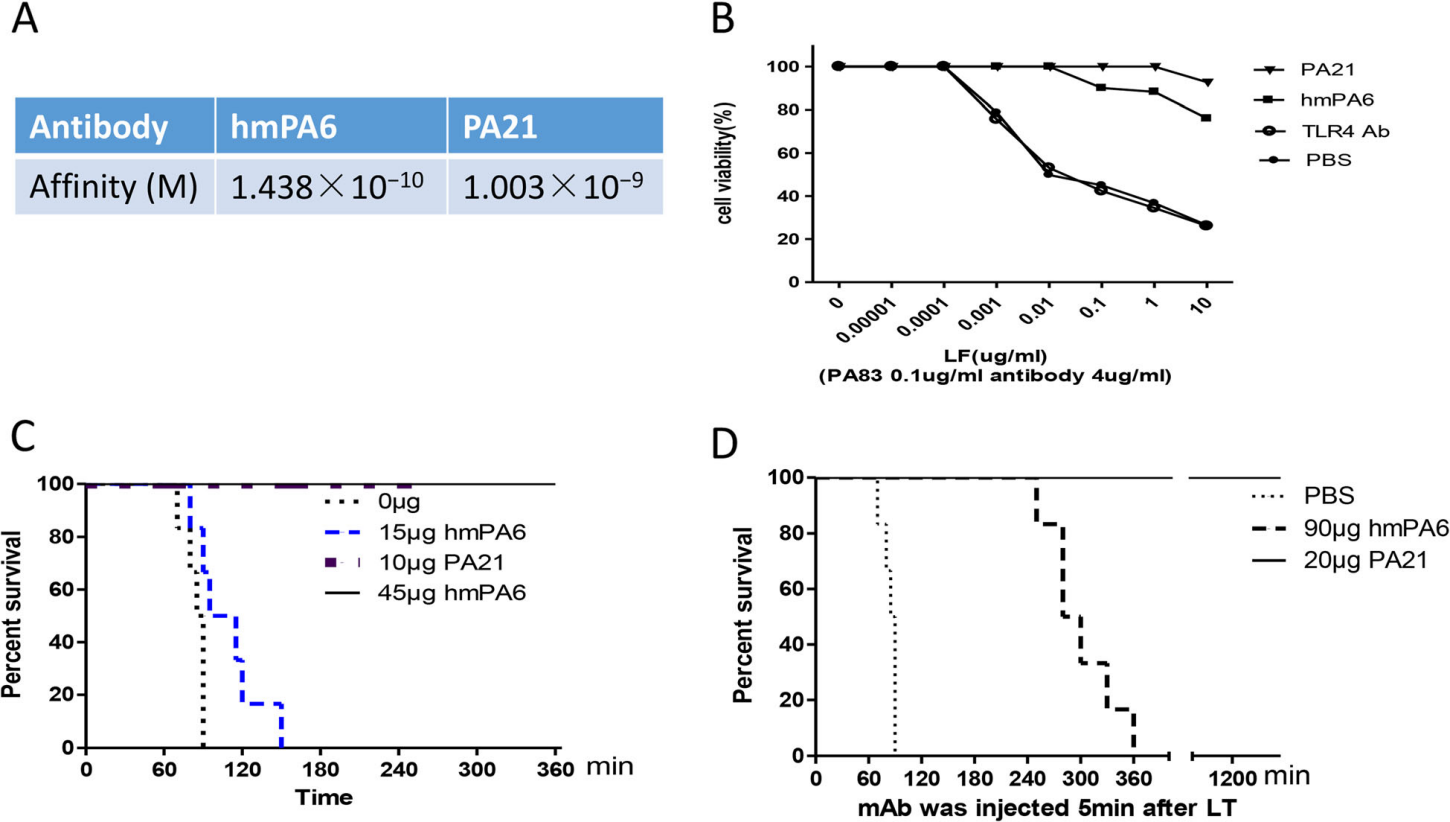
Affinity and neutralization function. **a** Affinity of hmPA6 and PA21. **b** Cell viability. Cell viability of hmPA6 and PA21 treated with lethal toxin in J774A.1 cell, 3 independent experiment. In vivo lethal toxin Neutralization Assay in F344 rats, **c** (mAb and lethal toxin injected together) and **d** (lethal toxin injected 5mins before mAb), *n* = 6. *Log-rank p* < 0.05

The affinity and neutralization assays were reported by our lab previously. Here, we compared these two antibodies, as shown in Fig. 1. Briefly, the equilibrium dissociation constants (Kd) of PA21 and hmPA6 were determined by BiaCoreX100 analysis. The affinity of hmPA6 and PA21 were 1.438 × 10–10 M and 1.003 × 10–9 M (Fig. 1a), respectively. J774A.1 cells were used to assess the ability of hmPA6 and PA21 to protect against LeTx. The antibody, PA83 and different concentration of LF were added to cells simultaneously. The cell viability indicated that the PA21 and hmPA6 could completely neutralize LeTx. At 10 μg/mL LF and 0.1 μg/mL PA83, > 80% of the hmPA6-treated cells and > 90% of the PA21-treated cells remained viable, while only 26% of the control IgG antibody-treated cells remained viable (Fig. 1b).

The in vivo neutralization test was performed on F344 rats. Antibody was injected via the tail vein simultaneously, before or after LeTx injection. In the simultaneously treated groups, all rats survived using 10 μg PA21 or 45 μg hmPA6 (Fig. 1c). In the antibody injected 5 min after LeTx groups, all rats survived using 20 μg PA21, while rats treated with hmPA6 did not (Fig. 1d).

### Inhibition of LF binding to PA63

#### Competitive ELISA

**Fig. 2:**
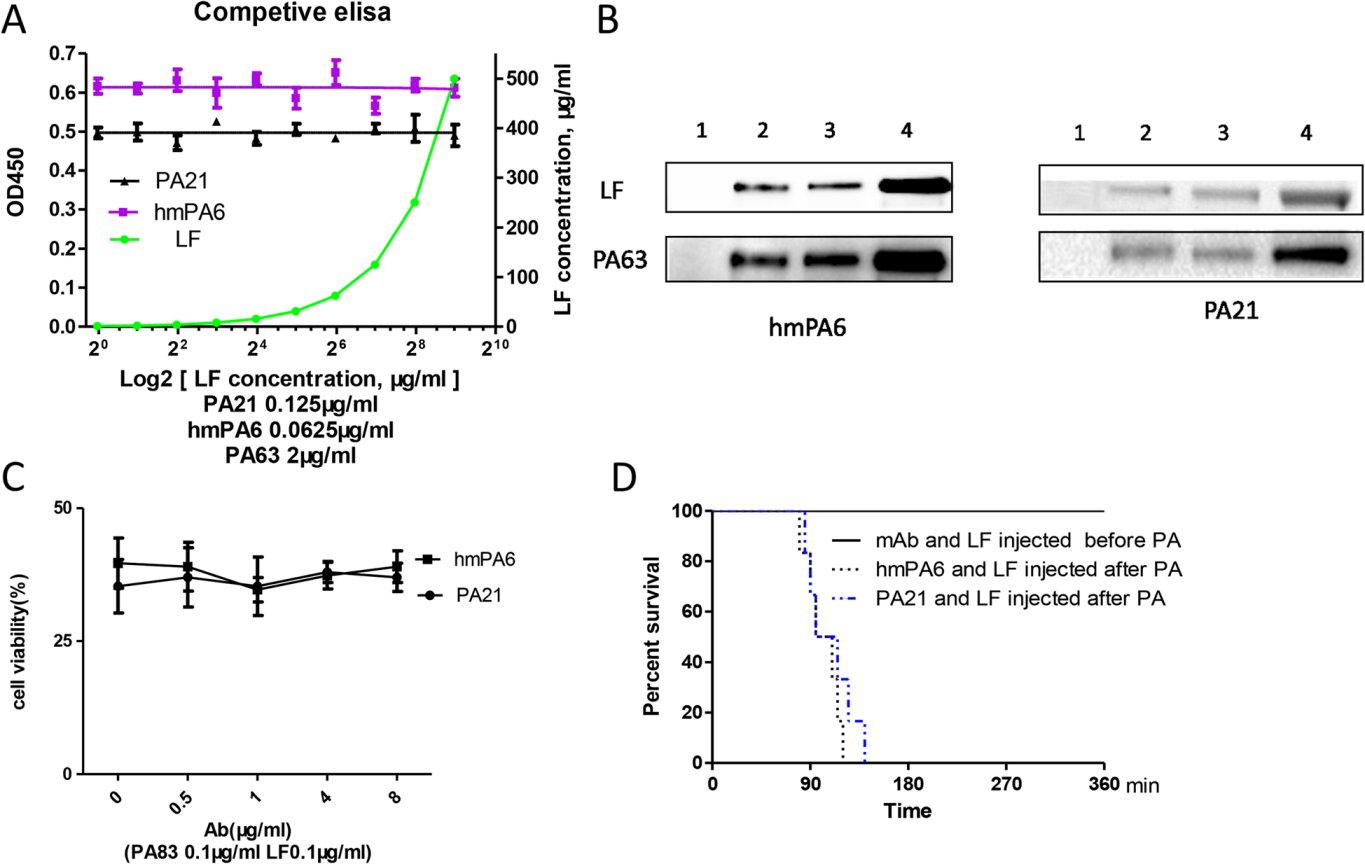
Inhibition of LF bond to PA assay. **a** Competitive ELISA, 3 independent experiments. **b** Co-IP. lane1, 2 μg antibody + 10 μg LF; lane2, 2 μg antibody + 1 μg PA63 + 10 μg LF; lane3, 2 μg antibody + 5 μg PA63 + 10 μg LF; lane 4, 2 μg antibody + 10 μg PA63 + 10 μg LF. **c** Cell viability of mAb added after PA, 3 independent experiments. **d** Rat survival, the mixture of LF and mAb were injected 15 min before or after PA, *n* = 6. *Log-rank p* < 0.05

The competitive ELISA was performed with LF, antibodies and PA63.The PA63 was coated to 96-well-ELISA plate in a concentration of 2 μg/mL. However, the OD450 of hmPA6 and PA21 was nearly identical, despite the increased concentration of LF (Fig. 2a).

#### Co-IP assay

The LF could be pulled down by hmPA6 and PA21 in the presence of PA63, while antibody alone could not pull down LF. Moreover, the amount of detected LF was positively correlated with detected PA63. Further, the hmPA6 and PA21 were displayed the same result (Fig. 2b).

#### Neutralizing assay

When the antibody was added 3 h after PA, even the antibody reached 8 μg/mL, the cells were not protected (Fig. 2c). Moreover, in rats test, the antibody could not protect rats when it injected after PA (Fig. 2d).

### Inteference of PA binding to cell receptor

**Fig. 3:**
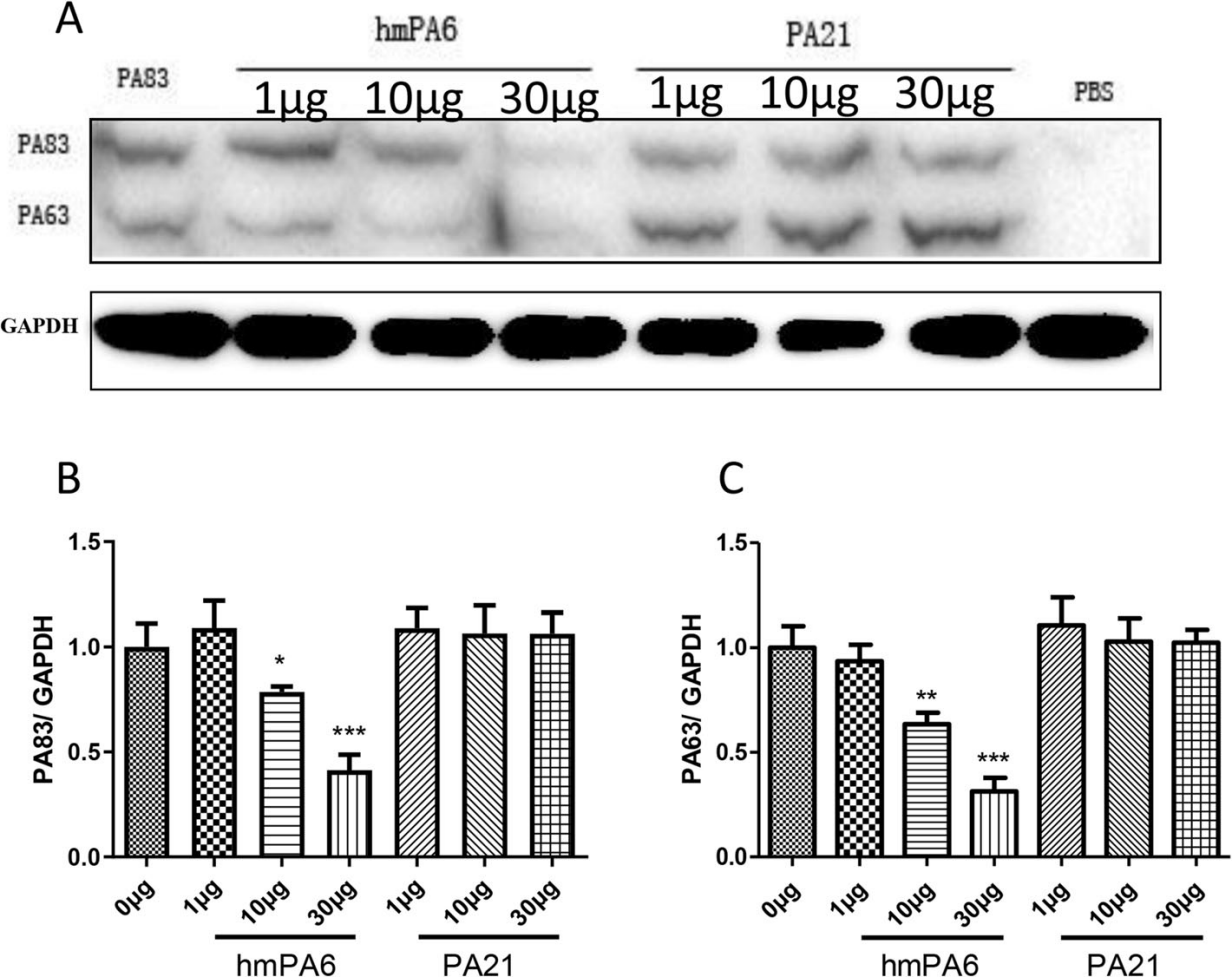
Inhibition of PA bond to receptors assay. **a** Western Blot. **b**&**c** Relative PA83 & PA63 intensity. *n* = 4, *P < 0.05 vs 0 μg group; ***P < 0.001 vs 0 μg group

Cell-bound PA83 (mostly cleaved to PA63) could be detected by anti-PA antibody. Briefly, 1 μg PA83 and different amounts of antibodies were added to J774A.1 cells. The cells incubated with PA83 alone served as a positive control, and non-treated cells were the negative control. The detected PA83 and PA63 displayed none significant difference among the different groups of increased PA21; however, the detected PA83 and PA63 were significantly decreased among the different groups of increased hmPA6 (Fig. 3).

### Prevention of PA cleavage by Furin

**Fig. 4:**
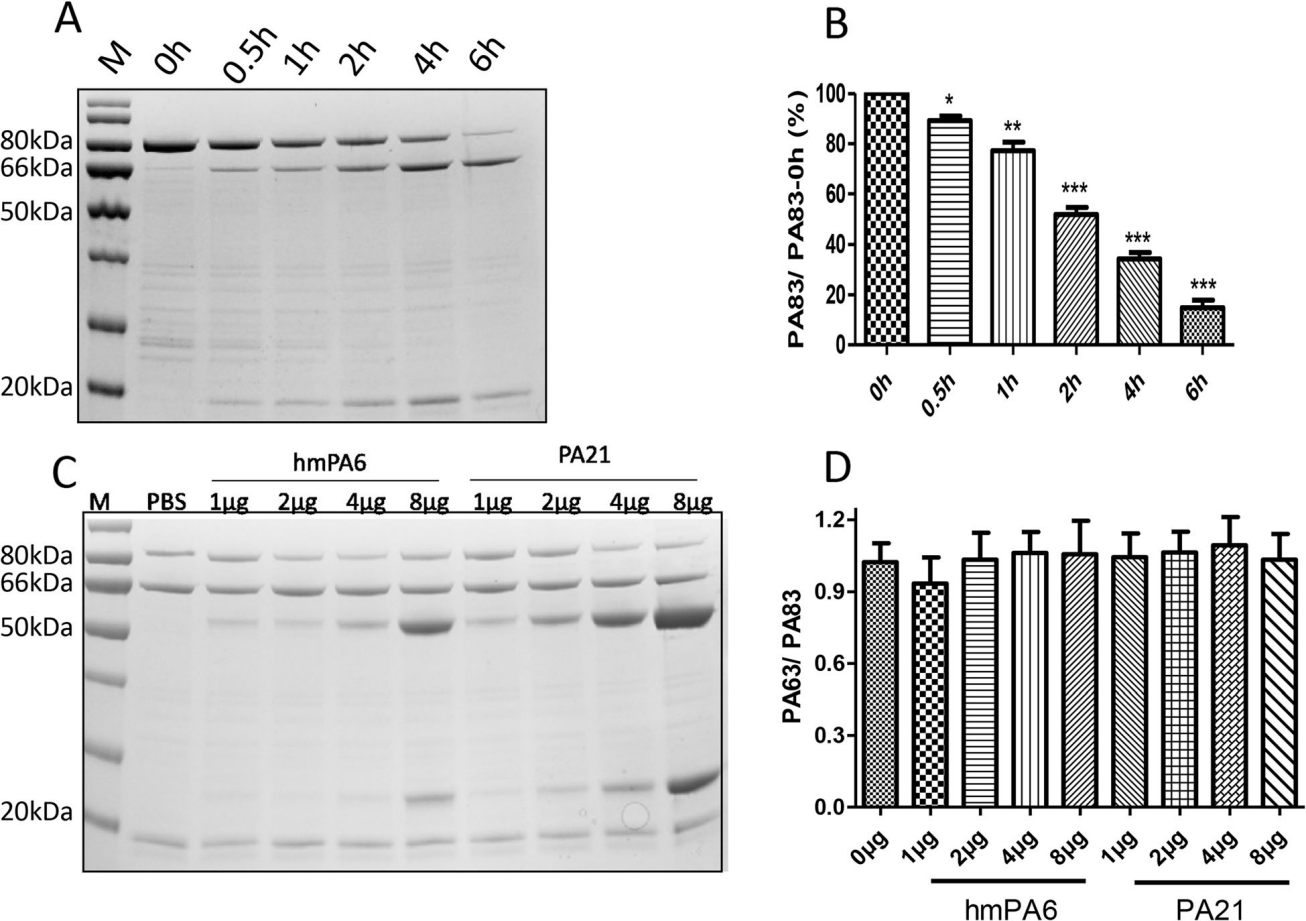
Inhibition of Furin cleaving PA test. **a** & **b** SDS-PAGE displays cleaved PA83 by Furin in different time. *n* = 4 **c** & **d** SDS-PAGE displays cleaved PA83 in different amount of mAb incubated with 25 μg PA and 1unit Furin. *n* = 4 *P < 0.05 vs 0 h group; ***P < 0.001 vs 0 h group

Furin was used to cleave PA83, the SDS-PAGE showed that when the cleaved time reached 6 h, it could cleave most PA83 (Fig. 4a and b). Different amounts of antibody were added to the mixture, and incubated for 6 h at room temperature. With the increased antibody, the amount of cleaved PA83 showed none significant difference in both antibodies (Fig. 4c and d).

### Recognition of different domains of PA

**Fig. 5:**
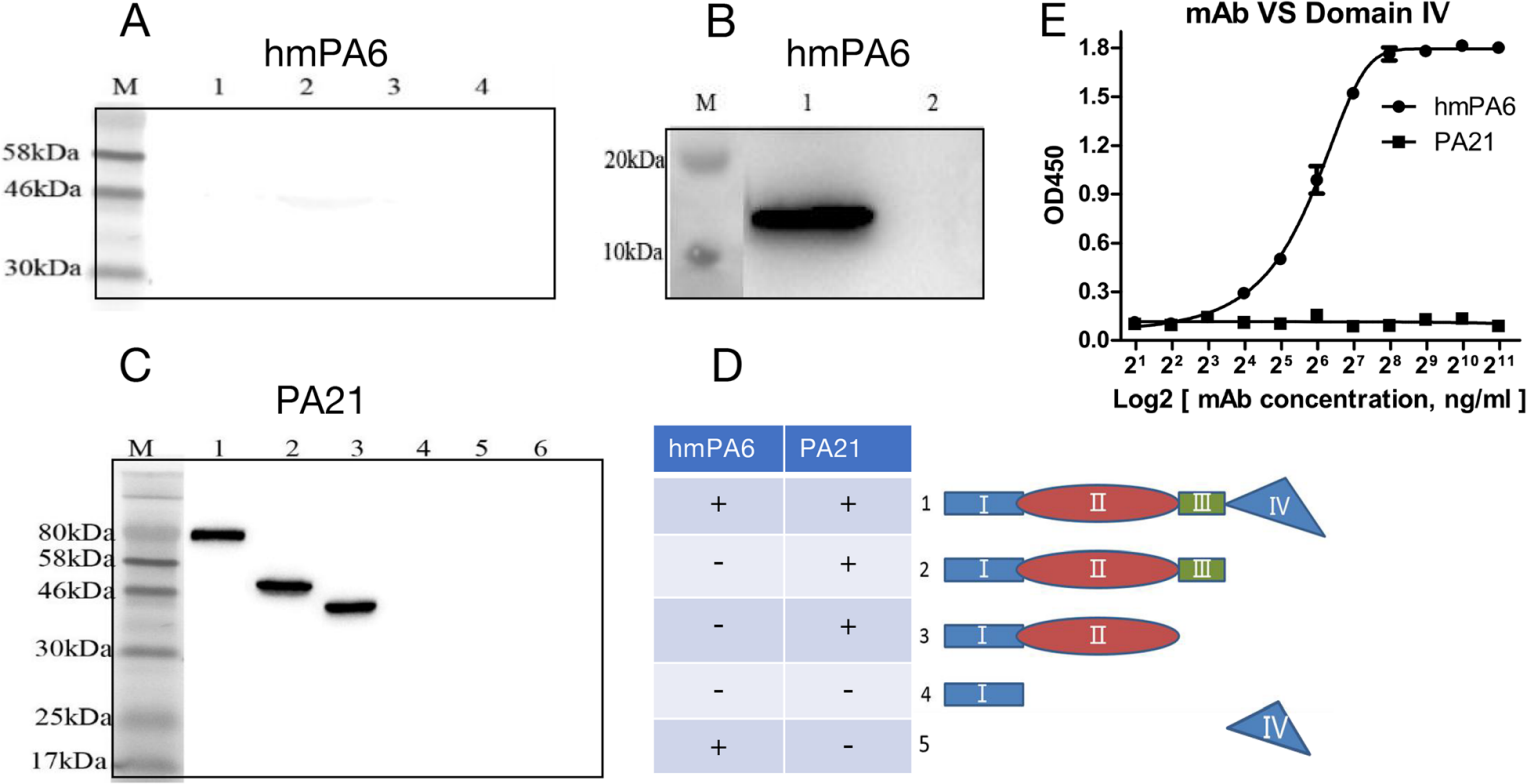
Epitope of mAbs. **a**&**b** Western blot of hmPA6. A, M, marker; lane 1, protein of domain Ib, II and III; lane 2, protein of domain Ib and II; lane 3, domain I of PA; lane 4, lysis of BL21- DE3. **b** M, marker; lane 1, domain IV of PA; lane 2, lysis of BL21- DE3. **c** Western blot of PA21. M, marker; lane 1,PA; lane 2, protein of domain Ib, II and III; lane 3, protein of domain Ib and II; lane 4, domain Iof PA; lane 5, domain IV of PA; lane 6, lysis of BL21- DE3. **d** sketch map, the protein of PA domains combination and the results of western blot. **e** ELISA of mAbs, 3 independent experiments

The domains of PA were constructed and expressed for western blot and ELISA. The hmPA6 could detect domain IV, however, it could not recognize protein of domainIb, IIand III (Fig. 5a and b). Moreover, hmPA6 recognized domain IVin a dose-dependent manner (Fig. 5e). Further, PA21 could detect proteins of domainIb, IIand protein of domain Ib, IIand III, however, it could not recognize domain IVand domainIalone (Fig. 5c).

## Discussion

In previous study, we constructed two effective anti-PA antibodies, PA21 and hmPA6. However, we did not elucidate their recognition epitope and their mechanism of neutralizing lethal toxin. Here, we compared these two antibodies and conducted an in-depth investigation to illuminate the mechanisms of their protective effects.

First, PA21 and hmPA6 could recognize PA with an affinity of 1.003 nM and 0.14 nM separately. They both had high affinity, yet, hmPA6 was better than PA21. Second, both PA21 and hmPA6 could neutralize lethal toxin in vitro. However, at an antibody concentration of 4 μg/mL, PA21 protected all cells at 1 μg/mL LF and 0.1 μg/mL PA83, while hmPA6 only protected 90% cells. Finally, the in vivo test showed the similar results. PA21 could protect all F344 rats alive at a concentration of 0.067 mg/kg (10 μg per rat), while hmPA6 required a concentration of 0.3 mg/kg (45 μg per rat). In all, although both PA21 and hmPA6 had high affinity and well neutralization function, the affinity and the protective effects were not positively correlated. PA21 was better in protecting cells and rats, while hmPA6 had higher affinity. As a result, we investigated the mechanism of the two antibodies.

According to previous reports, we studied the mechanism in the following aspects: inhibition of PA binding to cell receptors, interference with PA proteolytically cleavage by furin protease, inhibition of the heptamer assembly of the remaining PA63 on the cells, and interference with the PA63 heptamer combined LF/EF [22, 23]. Experiments were conducted to explicate whether both the two antibodies interfered with LF bond to PA63, such as competitive ELISA, Co-IP, etc. The competitive ELISA showed that PA21 (or hmPA6) and LF were combined with PA63 separately, they had no competitive relation with PA63. Co-IP, cells and rats’ protective tests displayed that PA21 (or hmPA6) could not inhibit LF binding to PA63. Consequently, we added PA and different amounts of PA21 (or hmPA6) into J774A.1 cells. After a 3 h incubation, we detected the PA (or PA63) binding to cell receptors. We found that PA21 had no effect on PA binding to cell receptors. However, the detected PA83 and PA63 were decreased among the different groups of increased hmPA6. Consistent with these findings, the hmPA6 cell western blot results were similar to the IHC of the lung tissue mentioned in our previous study [19]. Therefore, hmPA6 must inhibit PA binding to cell receptors. Next, we incubated furin protease, PA and different amounts of PA21 (or hmPA6) together. These two antibodies could not interfere PA cleaving to PA63 and PA20. By these experiments, we concluded that hmPA6 inhibited PA binding to cell receptors. Further we speculated that PA21 inhibited PA63 assembling to heptamer.

Furthermore, we reconstructed PA into different fragments: fragment containing only domain IV; fragment containing only domainI; fragment containing domain Ib, II and III; and fragment containing domain Ib and II. The western blot and ELISA experiments revealed that hmPA6 recognized domain IV, and PA21 recognized domain II. Several reports have already demonstrated that domain II and domain III are involved in heptamer and pore formation, while domain IV binds to the cellular anthrax toxin receptors [24–27]. Therefore, these epitope results were consistent with our mechanism results. In summary, hmPA6 recognized domain IV, thus domain IV of PA could not bind to cell receptors; PA21 recognized domain II (or domain II and III), thereby limiting oligomerization of PA63 on cells. The different mechanisms of the two antibodies caused different protective effects. PA21 showed better protective effects, as one molecular PA21 could inhibit seven or more molecular PA63 oligomerized to heptamer/octamer. However, one molecular hmPA6 perhaps only could inhibit one molecular PA bond to cell receptors.

## Conclusions

In this study, we first compared the affinity and neutralization function of hmPA6 and PA21. Further, we investigated the protective mechanisms of hmPA6 and PA21. Moreover, we characterized the domains of PA, which the two antibodies could recognize. We found that hmPA6 recognized domain IV, thus PA could not bind to cell receptors; and PA21 recognized domain II, thereby limiting heptamer oligomerization of PA63 in cells. The present investigation makes the two antibodies use in anthrax treatment or prophylaxis in the future clinical test more closely. Furthermore, they targeted on different epitopes indicate that they can used together.

## Data Availability

The datasets used and analyzed during the current study are available from corresponding author upon request.

## Abbreviations

EF: Edema factor
ELISA: Enzyme-linked immunosorbent assay
LF: Lethal factor
PA: Protective antigen
PBS: Phosphate buffer saline
SDS-PAGE: Sodium dodecyl sulphate Polyacrylamide gel electrophoresis
